# Evaluation of stripe rust resistance and genome-wide association study in wheat varieties derived from the International Center for Agricultural Research in the Dry Areas

**DOI:** 10.3389/fpls.2024.1377253

**Published:** 2024-04-09

**Authors:** Zhonghao Gao, Xin Wang, Yunxiang Li, Wanwei Hou, Xiaojuan Zhang

**Affiliations:** ^1^ School of Ecological and Environmental Engineering, Qinghai University, Xining, Qinghai, China; ^2^ Qinghai Academy of Agriculture and Forestry Science, Qinghai University, Xining, Qinghai, China; ^3^ National Crop Germplasm Resources Duplicate, Xining, Qinghai, China

**Keywords:** ICARDA, wheat varieties, stripe rust, 55K SNP, genome-wide association study

## Abstract

159 wheat varieties obtained from ICARDA, CYR32, CYR33 and CYR34 were used to evaluate the stripe rust resistance in this study. Seedling resistance was carried out in the green house at the two-leaf stage. Adult-plant resistance was carried out between 2022 and 2023 in Xining and Guide, respectively. A total of 24,151 high-quality SNP loci were obtained from a 55K SNP chip data. Genome-wide association study was carried out between SNP loci and stripe rust resistance. Seedling resistance screening revealed that 91.8% (146) of wheat varieties were resistant to CYR32 and CYR33, while only 49.7% (79) of wheat varieties were resistant to CYR34. Adult-plant resistance showed 153 (96.2%) germplasms represented resistance in 2022, while only 85 (53.4%) showed resistance in 2023. An association study using the 55K SNP chip data results combined with disease ratings of 159 materials at both the seedling and adult stages discovered 593 loci related to stripe rust resistance (P ≤ 0.0001). These loci exhibited contribution rates ranging from 11.1% to 18.7%. Among them, 71 were significantly related to resistance against CYR32 at the seedling stage, with a contribution rate of 12.7%-17.2%. Constituting the vast majority, 518 loci distributed across 21 chromosomes were significantly related to CYR33 at the seedling stage, with a contribution rate of 12.6%-18.7%. Fewer loci were found to be associated with disease resistance in adult plants. In E1 environment, a sole locus was detected on chromosome 2B with a contribution rate of 14.4%. In E2 environment, however, exhibited three loci across chromosomes 2B, 4A, and 7B with contribution rates ranging from 11.1% to 16.9%. A total of 68 multi-effect loci were significantly related to resistance against both CYR32 and CYR33 at the seedling stage, and one stable locus was significantly associated with stripe rust resistance at the adult plant stage.

## 1 Introduction

As one of the world’s most important cereal crops, wheat plays a central role in global food security (Juliana et al., 2017). Wheat stripe rust is prevalent in China but is especially troublesome in some provinces such as Qinghai, Guizhou, Shaanxi, Shanxi, Chongqing, and Sichuan (Dong et al., 2019). The disease is recognized as a significant threat to Chinese food security due to its wide distribution range, rapid transmission, and capacity to cause substantial yield losses (Wang et al., 2016; Dai et al., 2019). Since its initial discovery in Europe in 1777, the pathogen has been reported in over 60 countries (Line, 2002; Chen, 2005).Recently, scientists have reported a total of 86 (*Yr1*-*Yr86*) stripe rust resistance genes distributed across all chromosomes except chromosome 1A (Klymiuk et al., 2022). An additional 100 genes have been tentatively named but await official adoption. Moreover, researchers have mapped over 300 QTLs controlling wheat stripe rust resistance (Li et al., 2020; Feng et al., 2023; Zhu et al., 2023). Previous virulence analyses revealed that only *Yr5* and *Yr15* confer high resistance against races CYR32, CYR33, and CYR34 throughout the whole wheat growth period, while *Yr10*, *Yr24*, and *Yr26* are only effective against CYR32 and CYR33. *Yr9*, *Yr18*, and *Yr41* were susceptible against all three races during both the seedling and adult stages (Zeng et al., 2015; Wang et al., 2019). Previous research has tested thousands of wheat production varieties, reserve varieties, high-generation strains, and disease-resistant source materials for reactions to stripe rust at the adult plant stage. Many varieties previously considered resistant to wheat stripe rust were found to be susceptible to CYR34, indicating that the pathotype exhibits strong pathogenicity and high parasitic potential. These findings suggested that CYR34 posed a serious threat to wheat production in China (Huang et al., 2018). Furthermore, a new physiological race of stripe rust, tentatively named “TAS-6”, has been reported to overcome resistance conferred by *Yr5* (Zhang et al., 2020). Therefore, uncovering new sources of genetic disease resistance for use in wheat breeding is the key to ensuring global food security (Xu et al., 2020).

Plant breeders often rely on Genome-Wide Association Studies (GWAS), which allow them to analyze the genetic basis of traits at the population level by testing the significance of the association between genetic markers and phenotypic variations (Liu et al., 2017). GWAS has quickly become popular with plant researchers due to its faster results, greater breadth, and higher accuracy since 2001 (Thornsberry et al., 2001; Flint-Garcia et al., 2005). In 2006, researchers effectively used a GWAS to discover influential QLTs in wheat for the first time (Breseghello and Sorrells, 2006). The widespread use of the technology has broadened our understanding of the plant. To date, a total of 37 genes/QTLs, including 10 potential new QTLs, have been detected using 616 spring wheat varieties and breeding lines (Liu L. et al., 2020). New disease resistance loci have been discovered through GWAS by combining 9K SNPs with phenotypic data at the adult-plant stage (Maccaferri et al., 2015). In a previous association study utilizing 660K SNPs from 411 spring wheat lines grown over multiple years and environments, researchers identified TraesCS2B01G513100, a candidate gene that is associated with stripe rust resistance. The researchers then developed molecular markers to assist with disease resistance breeding (Wu et al., 2020). Based on 90K SNP chip data, a separate association study of 375 natural wheat varieties from home and abroad identified 26, 22, and 25 significantly associated SNP loci on the short arms of 2AS, 2BS, and 2DS in the second homology group, respectively. These loci can explain 4.61%-11.75% of the observed phenotypic variation (Zhang et al., 2019). Taken together, the results of these studies provide a basis for the cultivation of new stripe rust-resistant wheat varieties and offer guidance for the discovery of new genes.

ICARDA’s (the International Center for Agricultural Research in the Dry Areas) mission is to address challenges caused by harsh and ever-changing environments; and ICARDA’s breeding program also emphasizes the importance of utilizing varieties with local adaptability, as well as the necessity of plant biodiversity for human survival; in addition, ICARDA preserves thousands of wheat crop resources, which provides necessary conditions for us to introduce wheat materials from ICARDA and cultivate new varieties (Lu, 2022). With the continuous development of molecular markers and the emergence of new technologies, novel stripe rust resistance genes are becoming more plentiful and more accessible for wheat breeders (Luo et al., 2010). Various molecular marker techniques have been adopted by researchers to analyze stripe rust resistance and to discover new resistance loci (Bulli et al., 2016; Liu et al., 2018). Recently, there is little molecular-based research regarding agronomic traits, disease resistance, and stress resistance in introduced wheat varieties in Qinghai Province. In this study, we conducted a GWAS using introduced ICARDA wheat varieties and 55K SNP to uncover potentially significant disease resistance loci in both wheat seedlings and adult plants, which offered a theoretical reference for the stripe rust resistance of ICARDA wheat varieties and excavating new stripe rust resistance genes or QTLs.

## 2 Materials and methods

### 2.1 Test materials

159 wheat varieties derived from ICARDA were obtained and designated as ICARDA001-ICARDA159 (**Supplementary Table 1**). CYR32, CYR33, and CYR34 were provided by the wheat stripe rust breeding laboratory of Northwest A&F University.

### 2.2 Disease resistance evaluation at the seedling stage

To assess disease resistance in wheat seedling stage, 10 seeds of a wheat variety were sown in a container and grown under suitable conditions. Once the plants reached the two-leaf stage, they were uniformly sprayed with a 1:100 ratio of Tween to water. Next, each physiological race of stripe rust was mixed with talc in a 1:20 ratio and shaken onto the test varieties. After the wheat leaves were completely diseased, the infection type was rated according to Line and Qayoum’s 9-level reaction pattern.

### 2.3 Disease resistance evaluation at the adult-plant stage

The stripe rust resistance of adult wheat was evaluated between 2022 and 2023 in experimental fields located in Xining and Guide of Qinghai Province. In 2022, trials in Xining and Guide were designated E1 and E2, respectively, and were inoculated with a mixture of CYR32 and CYR33. In 2023, trials in Xining and Guide were designated as E3 and E4, respectively, and were inoculated with a mixture of CYR32 and CYR34. Wheat lines were scored by recording the number of rust lesions on each leaf during the height of infection.

### 2.4 Genotypic analysis

The genome of 159 wheat varieties was analyzed using a 55K SNP chip developed by the Affymetrix Axiom platform, provided by ZhongYuJin Marker (Beijing) Biotechnology Co., Ltd. Affymetrix Axiom Analysis Suite software (Thermo Fisher) was employed for preliminary screening and genotyping. A total of 53,063 loci were obtained from the raw data, following criteria of DQC (Data Quality Control) of ≥0.82 and CR (Call Rate) of ≥0.95 (https://datadryad.org/stash/share/aphTKrewmqNQnQgdj7vgUlw7I8jPMCT0GYiCyDNneSw). VCFtools software (Danecek et al., 2011) was used for site filtering with a missing rate of >20% and an MAF (Minor Allele Frequency) <0.05, resulting in 24,151 high-quality SNP sites. The marker density map for the obtained high-quality SNP loci was plotted using the CMplot package in the R suite.

### 2.5 Genetic diversity and population structure

The genetic diversity was calculated with PowerMarker V3.25 software (Liu and Muse, 2005) using the obtained 24,151 high-quality SNP sites. These calculations adhered to the PIC (Polymorphic Information Content) parameter, represented by PIC=1-Σ(P_ij_)^2^ (P_ij_ represents the frequency of the j^th^ allele at the i locus). Plink 1.9 software (Purcell et al., 2007) was used to perform LD filtering on SNP sites, resulting in 2,821 markers. Admixture software (Alexander et al., 2009) was used to analyze the population structure. The range of K values for the subgroups was preset to be 2-15, and the average value was calculated based on six repetitions. The average Cross-Validation (*CV*) value was calculated for each K value, and the K value associated with the lowest *CV* value was selected as the optimal subgroup number. Finally, a subgroup map was drawn using R software.

### 2.6 GWAS of stripe rust resistance

A GWAS was conducted on the 159 wheat varieties using 24,151 high-quality SNPs, phenotypic stripe rust resistance data collected at both the seedling and adult plant stages, a kinship matrix (K-matrix) generated by Tassel 5.0 software (Bradbury et al., 2007) and a Q-matrix created by Admixture.

## 3 Results and analysis

### 3.1 Stripe rust resistance identification at seedling and adult-plant stages

**Figure 1:**
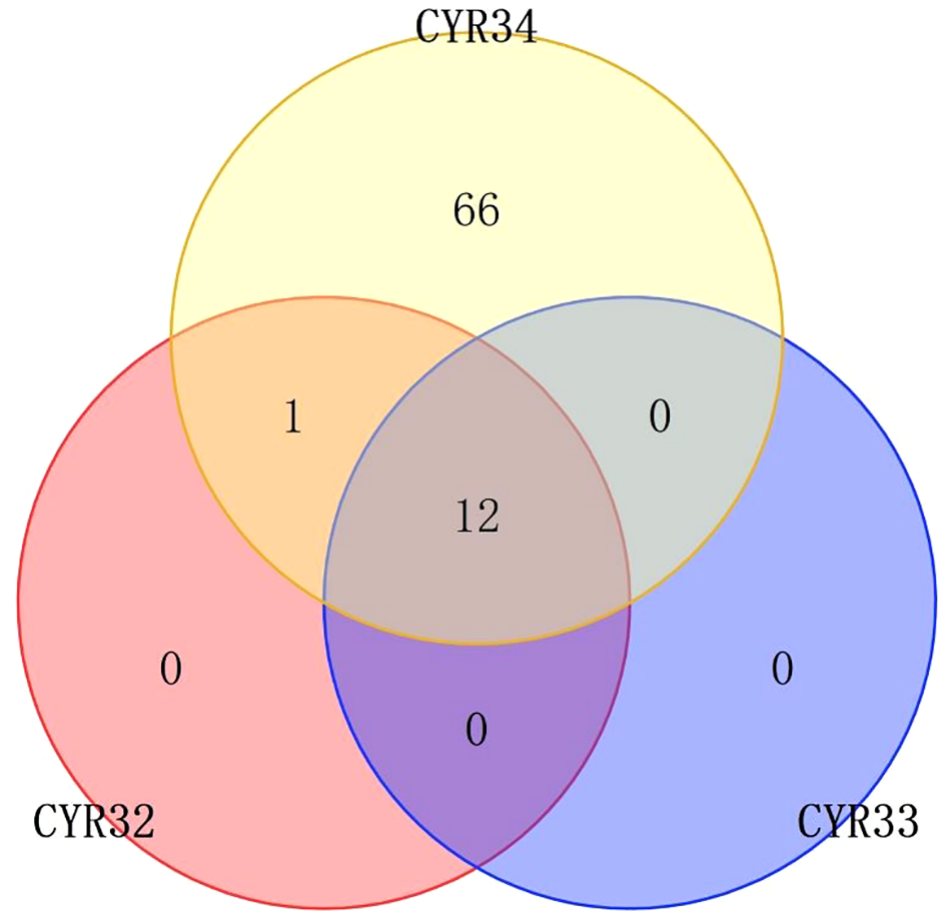
Venn plot of resistance distribution of wheat materials to CYRThe numbers in the figure represent the number of wheat materials that are resistant to this CYR, and the overlap represents the number of wheat materials that are resistant to both two or three CYR species.

During disease resistance screening of wheat seedlings, 21 (13.2%) varieties showed immunity to CYR32, while 125 (78.6%) varieties showed high resistance to CYR32, the remaining 13 (8.2%) lines showed susceptibility to rust stripe race CYR32, including IR002, IR017, IR037, IR053, IR055, IR061, IR073, R077, IR078, IR125, IR128, IR135, and IR149. For CYR33, 18 (11.3%) varieties showed immunity, 128 (80.5%) wheat materials showed high resistance, 1 (0.6%, IR133) varieties showed moderate resistance, and 12 (7.6%) varieties showed susceptibility (except for IR135 among the 13 materials mentioned above). For CYR34, 11 (6.9%) materials showed immunity, 68 (42.8%) materials showed high resistance, 1 (0.6%, IR107) material showed moderate resistance, and 79 (49.7%, including the 13 materials aforementioned) materials showed susceptibility, and most of them are highly susceptible to diseases (**Supplementary Table 2**; **Figure 1**). With the pervalent race of CYR34, most wheat varieties lost resistance at the seedling stage.

For the stripe rust evaluation of the adult plant stage in Xining, under the E1 environment, 6 materials (3.8%, IR017, IR055, IR125, IR128, IR135, and IR149) showed susceptibility, 111 materials (69.8%) showed high resistance, and 42 materials (26.4%) showed moderate resistance; in the E3 environment, 74 (46.5%) materials showed susceptibility, of which 68 showed resistance in 2022, 72 (45.3%) showed high resistance, and 13 (8.2%) showed moderate resistance. Compared with the E1and E3 environment, there were 50 materials (31.4%) with enhanced disease resistance in the E3 environment, 12 materials (7.5%) with unchanged resistance, and 23 materials (14.5%) with reduced resistance (**Supplementary Table 3**).

For the identification results of the adult plant stage in Guide, under E2 environment, 6 (3.8%, the same as E1 environment) materials showed susceptibility, 113 (71.1%) materials showed high resistance, and 40 (25.2%) materials showed moderate resistance; in the E4 environment, 74 (45.3%, similar to the E3 environment) materials showed susceptibility, of which 68 materials showed resistance in 2022, 66 (41.5%) materials showed high resistance, and 19 (11.9%) materials showed moderate resistance. Compared with E2 and E4 environment, there were 49 materials (30.8%) with enhanced resistance in the E4 environment, 12 materials (7.5%) with unchanged resistance, and 24 materials (15.1%) with reduced resistance (**Supplementary Table 3**).

### 3.2 Genotype analysis

**Figure 2:**
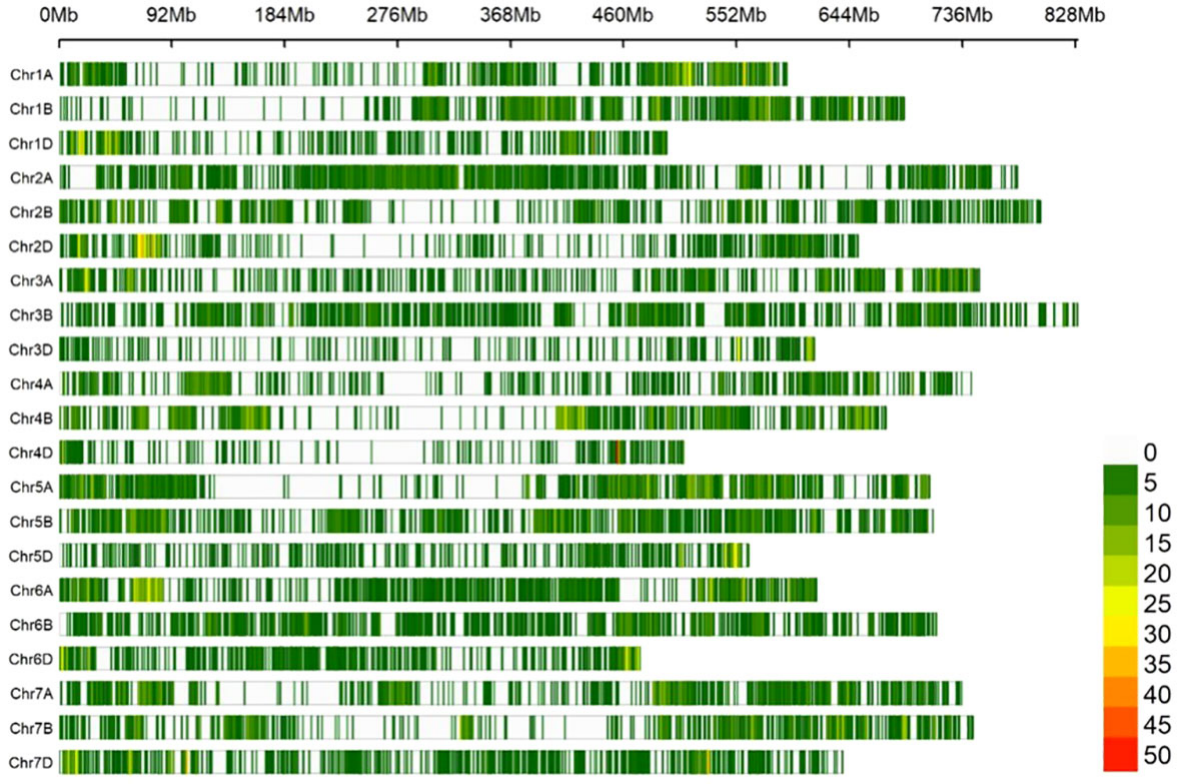
SNP density map. Indicates the number of SNPs per 1Mb of length on each chromosome, and different colors indicate different SNP densities.

The SNP density map illustrated a non-uniform distribution of 24,151 high-quality SNP loci across three genomes and 21 chromosomes, there was a portion of fragments without SNP markers, and the density was almost entirely concentrated at 5-15 loci/Mb. Among them, the SNP density on chromosome 3D was the highest at 1.7424/Mb, followed by 4D chromosome at 1.4263/Mb, and chromosome 6A has the lowest density at 0.3982/Mb; the number of SNPs on chromosome 4B was the highest, with 1675, followed by chromosome 5B with 1652, and the smallest was chromosome 4D with only 357. There were 9,338 (38.7%), 9,865 (40.8%), and 4,948 (20.5%) polymorphisms identified in genomes A, B, and D respectively. The overall polymorphism ratio was 45.5% (2,4151/53,063), with the highest occurrence in genome B, followed by A and then D. Among them, chromosome 2A possessed the highest PIC value of 0.3526, followed by chromosome 4A with a PIC value of 0.3467. The lowest PIC value was chromosome 3B with 0.2433; the PIC value of the genome A was the highest at 0.3264, followed by the B at 0.2999, and D chromosome at 0.2937. The average PIC value of all chromosomes is 0.3067 (**Table 1**; **Figure 2**).

**Table 1:** SNP markers and polymorphisms of 159 wheat materials.

Chromosome	Number of markers	Length (Mb)	Density of markers	PIC	Genetic diversity (GD)
1A	1221	593.24	0.4859	0.3053	0.3714
1B	1362	688.35	0.5054	0.2771	0.3330
1D	818	495.23	0.6054	0.3100	0.3781
2A	1540	780.70	0.5069	0.3526	0.4387
2B	1175	799.25	0.6802	0.3318	0.4119
2D	951	650.88	0.6844	0.3457	0.4347
3A	1163	749.87	0.6448	0.3099	0.3802
3B	1373	829.73	0.6043	0.2433	0.2843
3D	418	615.46	1.4724	0.2595	0.3088
4A	976	743.32	0.7616	0.3467	0.4385
4B	1675	673.47	0.4021	0.3098	0.3769
4D	357	509.18	1.4263	0.2970	0.3640
5A	1590	709.22	0.4461	0.3167	0.3905
5B	1652	711.84	0.4309	0.3166	0.3854
5D	618	561.92	0.9093	0.2743	0.3276
6A	1550	617.25	0.3982	0.3406	0.4143
6B	1335	714.62	0.5353	0.3118	0.3806
6D	765	473.30	0.6187	0.2928	0.3551
7A	1298	735.46	0.5666	0.3132	0.3821
7B	1293	744.39	0.5757	0.3087	0.3776
7D	1021	638.19	0.6251	0.2767	0.3295
A genome	9338	4929.06	0.5278	0.3264	0.4023
B genome	9865	5161.65	0.5232	0.2999	0.3642
D genome	4948	3944.16	0.7971	0.2937	0.3568
Total	24151	14034.87	0.6161	0.3067	0.3744

### 3.3 Genetic diversity and population structure

The analysis of genetic diversity (GD) revealed an average GD of 0.3744 among the 159 wheat lines, with variations ranging from 0.284 3to 0.4387. Genome A demonstrated the highest GD, followed by B and D. Among all chromosomes, the 2A chromosome had the highest GD value of 0.4387, followed by the 4A chromosome with a GD value of 0.4385, and the 3B chromosome had the lowest value of only 0.2843.

**Figure 3:**
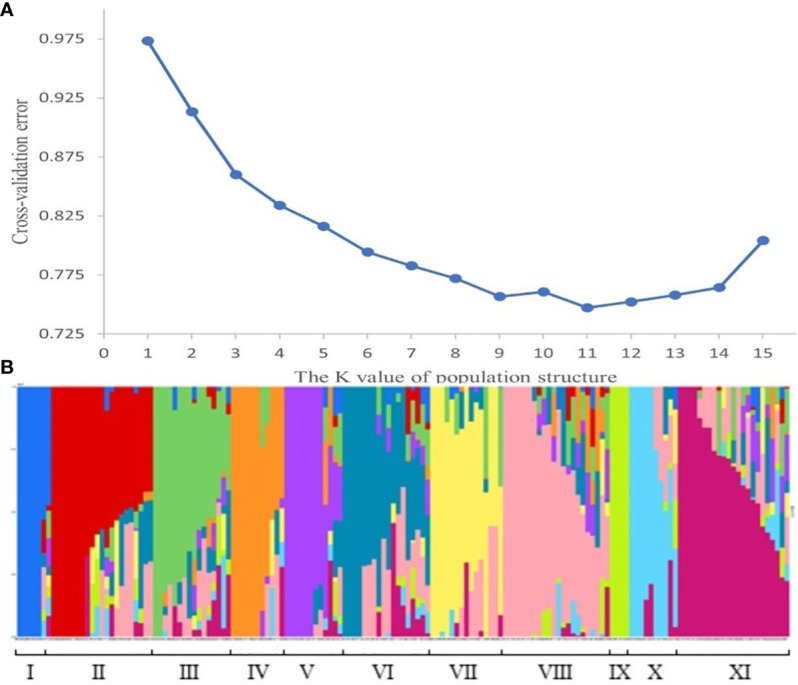
Population structure. **(A)** represents the CV value for the number of subgroups. The abscissa is the K value of the population structure, and the ordinate is the CV value corresponding to the K value, and the figure shows that when K=11, the CV value is the smallest, that is, 11 is the optimal number of subgroups. **(B)** represents the composition of each subgroup. Each histogram represents a wheat material. The color and proportion of this histogram indicates which subgroup the material belongs to and what the proportion of descent it belongs to.

The population structure analysis of the 159 wheat materials revealed that at the minimum *CV* value, K=11, suggesting that the materials could be divided into 11 subgroups. The Q-matrix generated when K=11 was employed as the covariate in the subsequent association study. The subgroups were comprised of the following: subgroup I with 7 (4.4%) varieties; subgroup II with 21 (13.2%) varieties; subgroup III with 16 (10.1%) varieties; subgroup IV with 11 (6.9%) varieties; subgroup V with 12 (7.6%) varieties; subgroup VI with 18 (11.3%) varieties; subgroup VII with 15 (9.4%) varieties; subgroup VIII with 22 (13.8%) varieties, subgroup IX with 4 (2.5%) varieties; subgroup X with 10 (6.3%) varieties; and subgroup XI with 23 (14.5%) varieties (**Figure 3**).

### 3.4 Association study

**Figure 4:**
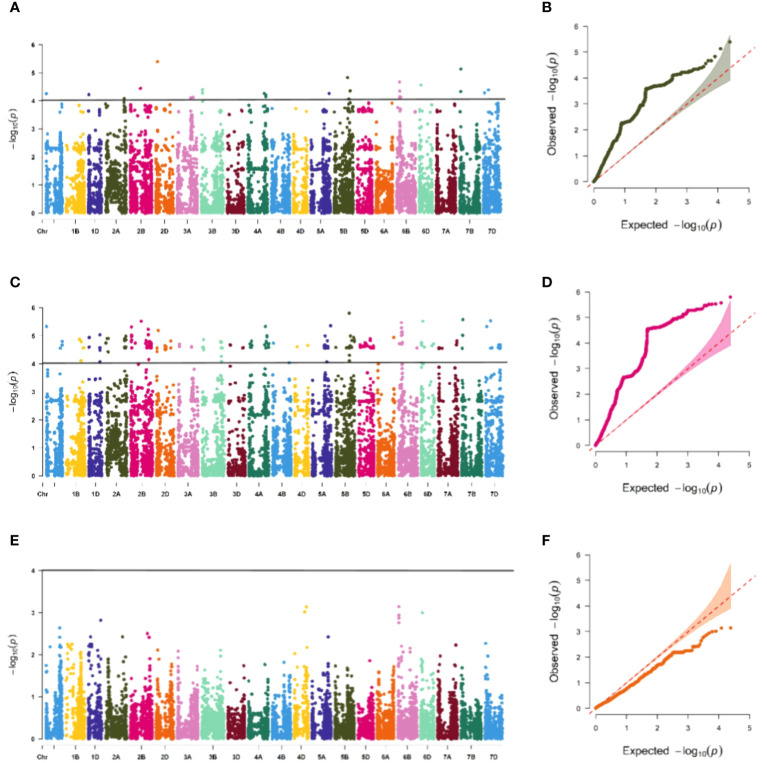
Association study of disease resistance at seedling stage. The Manhattan plot shows the degree of association between SNP loci and traits on each chromosome, and the stronger the association, the higher the height in the figure (left), and the QQ plot shows the consistency between the observed value (ordinate) of P value (ordinate) and the expected value of P value (abscissa), with a significant locus above the dashed line and no significant locus below the dotted line (right). **(A, B)** are Manhattan plot and QQ plot of CYR32 identification, respectively, and there are loci significantly related to CYR32 on most chromosomes, **(C, D)** are Manhattan plot and QQ plot of CYR33 identification, respectively, and there are loci significantly related to CYR33 on all chromosomes, and **(E, F)** are Manhattan plot and QQ plot of CYR34 identification, respectively, and none of the tested materials contain loci that are significantly related to CYR34.

**Figure 5:**
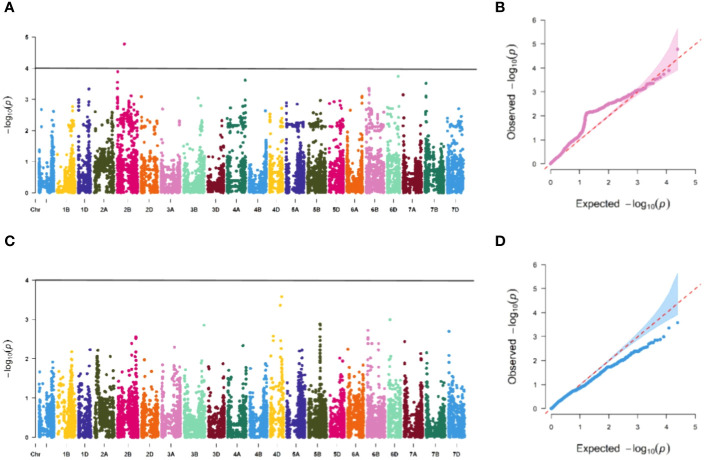
Association study of disease resistance at adult-plant stage in Xining **(A, B)** are the Manhattan plot and QQ plots of E1, respectively, and only one chromosome has a significant correlation locus, and **(C, D)** are the Manhattan plot and QQ plot of E3 identification, respectively, and none of the tested materials contain significant correlation.

**Figure 6:**
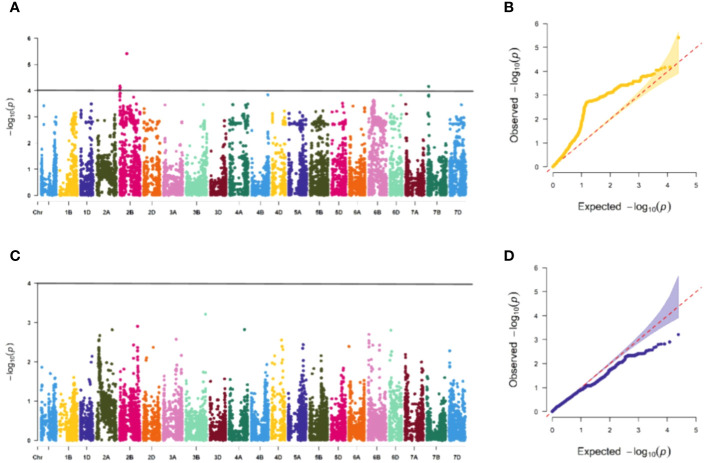
Association study of disease resistance at adult-plant stage in Guide **(A, B)** are the Manhattan plot and QQ plots of E2, respectively, with a total of 3 significant loci distributed on the 3 chromosomes, and **(C, D)** are the Manhattan plot and QQ plot of E4 respectively, and none of the test materials contain significant correlation.

The association study of ICARDA wheat varieties using 24,151 high-quality SNP loci and phenotypic data identified 593 loci associated with stripe rust resistance, contributing rates ranging from 11.1% to 18.7%. During the seedling stage, 71 loci were found to be related to stripe rust race CYR32. These loci were distributed across chromosomes 1A, 1D, 2A, 2B, 2D, 3A, 3B, 4A, 5A, 5B, 6B, 6D, 7B, and 7D, with contribution rates ranging from 12.7% to17.2%. With the exception of AX-109949596, AX-108783340, and AX-111778082, these loci were also involved in resistance against CRY33 with a contribution rate of 12.6%-17.2%, suggesting that they were multi-effect loci. An additional 518 loci were related to CYR33 and were distributed across all chromosomes with contribution rates of 12.6%-18.7%. In the association study of adult plants, we identified one stable locus, AX-109318462, on chromosome 2B with contribution rates of 14.4%-17.0% in E1 and E2 (**Figures 4**–**6**; **Supplementary Table 4**). An additional three loci on chromosomes 2B, 4A, and 7B were detected in E2, with contribution rates ranging from 11.1% to 16.9%. No significant loci were detected in E3 and E4. According to the results of the QQ plot, there were loci that were significantly associated with the stripe rust CYR32 and CYR33, E1 and E2 environments in all loci. Although there was a deviation between the observed P value and the expected P value in the QQ plot, which may be due to errors in statistics during the seedling and adult stage phenotype periods by manpower, it can have a certain impact on the results, but overall it can correspond to the results in the Manhattan plot.

## 4 Discussion

With the global warming and extreme weather, changes in temperature and precipitation have made the harm of stripe rust increasingly aggravated. A recent stripe rust pandemic was reported in 2017, and experts believed that the pandemic was caused by the year’s extremely warm winters, heavy spring rains and a lack of resistance diversity in the main wheat breeding areas (Zhang, 2022). In 2020, wheat stripe rust was included in the list of first-class crop diseases and pests by China, and how to prevent and control effectively is also a key research topic in the world. Therefore, plant protection experts have been constantly updating and improving the management system and technology for many years, but due to the continuous evolution of stripe rust races, abnormal global climate change, etc., the research on stripe rust and disease-resistant varieties will be continuous. At present, the basic and long-term effective means of stripe rust control is the breeding of disease-resistant varieties. However, there is a co-evolution between stripe rust and the host, new types of pathogenic physiological races continue to appear, and the resistance of most wheat varieties in China will be susceptible after 3~5 years of large-scale application, resulting in a disease pandemic (Chen et al., 2013). Due to its special geographical location, Qinghai Province is one of the main summer epidemic areas of wheat stripe rust in China. Because of the influence of climatic conditions, spring wheat is the main wheat plantation, and a large number of stripe rust fungus sources can be found in late spring wheat varieties over summer (Zhang and Hou, 2022). At the same time, since wheat is the main food crop in Qinghai Province, new disease resistance genes were obtained after disease resistance identification and genome-wide association study of wheat materials, which were then introduced into Qinghai Province to enrich the wheat germplasm resources and lay a certain foundation for further wheat breeding research.

### 4.1 Disease resistance identification

In the screenings of 159 wheat varieties at the seedling stage and during a two-year, two-environment adult field trial, we identified 146 (91.8%) lines that were resistant to both CYR32 and CYR33, while only 80 (50.3%) were resistant to CYR34. These results are consistent with previous studies and suggest that most of the introduced wheat varieties had good resistance to CYR32 and CYR33, indicating that these varieties contained genes, gene combinations and even new genes that could effectively resist CYR32 and CYR33, which could effectively prevent the epidemic of these two epidemic races in the future, and the current materials did not have enough resistance to the new toxic race CYR34 (Zhang et al., 2022). This may be attributed to the early application of CYR32 and CYR33 promoting resistance in wheat (Zeng et al., 2015). Yao conducted a preliminary study on the overwintering conditions of wheat stripe rust in the eastern wheat area of Qinghai, and the results showed that the amount of bacteria before winter was the primary factor affecting the overwintering of wheat stripe rust (Yao et al., 2014). Therefore, the utilization of disease resistance during the whole growth period is of great significance for the prevention and control of stripe rust fungus in Qinghai wheat. Subsequently, these 159 materials can be used for molecular testing to obtain the disease resistance genes that may be contained in them, so as to provide a basis for the development of new varieties.

### 4.2 Association study of disease resistance

The PIC variation of the 159 materials ranged from 0.243 to 0.353, with an average of 0.307. This indicates that the SNP markers were moderately polymorphic, which is consistent with previous studies (Kumar et al., 2020; Liu YK. et al., 2020). Additionally, the genetic diversity ranged from 0.284 to 0.439, with an average of 0.374. This suggests a relatively concentrated genetic background between the breeds, aligning with previous research (Cao et al., 2015; Li et al., 2019). An analysis of the 55K SNP chip data from 159 ICARDA wheat varieties combined with the phenotypic stripe rust resistance data revealed that genome B contained the highest quantity of polymorphisms, while genome D exhibited the least. These findings reflect the works of Zhang (Zhang et al., 2016) and Tehseen (Tehseen et al., 2021). A total of 593 significant loci were detected, 68 of which were significant in conferring resistance against both CYR32 and CYR33. These loci are distributed across chromosomes 1A, 1D, 2A, 2B, 2D, 3A, 3B, 4A, 5A, 5B, 6B, 6D, 7B, and 7D. The analysis of stripe rust resistance at the adult-plant stage identified locus AX-109318462 on chromosome 2B as the sole locus associated with resistance in both E1 and E2. Subsequently, referring to the IWGSC_RefSeq_v1.0, the loci within the 5Mb interval on the physical map were considered as one significant locus. Using the QTL genetic map of wheat stripe rust resistance constructed by Cheng (Cheng et al., 2019) and the comparison diagram by Yao (2022), the 69 loci were compared and analyzed. It was found that 9 of these 69 loci may be newly discovered loci, namely AX-110974432 (3A), AX-110447030 (3A) AX-108736767 (3A), AX-89776892 (3A), AX-110653920 (5B), AX-109997800 (5B), AX-95252437 (6D), AX-86163952 (7B), AX-110962394 (7B) (the chromosome where the locus is located is indicated in parentheses); the remaining loci are the same or similar to the reported QTL or *Yr* gene positions. The two loci located on chromosome 5B partially coincided with Qyr.pd-5B.1 between the markers Xbarc275 and XIWA2095, and were very close to the reported QTLs related to stripe rust disease type and QTLs associated with infection type, respectively, so it was speculated that there may be genes related to stripe rust type or reactive type or both in the vicinity of these two loci, and two loci located on chromosome 7B were close to the *Yr63* gene on this chromosome, *Yr63* is located at a hot spot known for against pests and diseases in plants and animals, which was enriched with multi-nucleotide-binding and leucine rich repeat (NLR) and kinase domain encoding genes (Mackenzie et al., 2023), it is speculated that it is possible to discover new defense genes near this site.

## 5 Conclusion

In this study, 159 ICARDA wheat varieties were tested for stripe rust resistance. The majority of these lines were resistant to races CYR32 and CYR33, suggesting their potential for widespread cultivation in Qinghai Province. These findings further enrich the repository of disease-resistance genes deployed in local wheat breeding in Qinghai Province. During the association study using 55K SNP chip data and phenotypic disease resistance data from both adult and seedling stages uncovered a total of 593 loci, 589 of which were related to CYR32 and CYR33 and distributed across 21 chromosomes. Four loci were specifically related to the adult plant stage and were distributed on chromosomes 2B, 4A, and 7B. Additionally, we identified 68 multi-effect loci which were associated with disease resistance at the seedling stage. Only one stable locus was related to disease resistance at the adult-plant stage. After analysis and comparison, 60 of the 69 loci were the same or close to the reported QTL or *Yr* gene positions, which were likely to be the same locus, and the specific relationship needed further study; while 9 loci were relatively far apart, which can exclude the possibility of being the same locus, they may be newly discovered loci in this study and required further precise localization research. This study layed the foundation for preventing wheat stripe rust in China by serving as a theoretical reference for the utilization of novel resistance loci and by providing guidance for the discovery of new resistance genes.

## Data availability statement

The data presented in the study are deposited in the Dryad Digital Repository, DOI number: 10.5061/dryad.4xgxd25ht, and the dataset has already been published.

## Author contributions

ZG: Data curation, Formal analysis, Investigation, Software, Writing – original draft. XW: Data curation, Formal analysis, Investigation, Software, Writing – original draft. YL: Formal analysis, Investigation, Software, Writing – original draft. WH: Investigation, Supervision, Writing – review & editing. XZ: Conceptualization, Funding acquisition, Methodology, Project administration, Resources, Supervision, Validation, Writing – review & editing, Data curation, Investigation.
