# Supplementary material for: Evaluation of stripe rust resistance and genome-wide association study in wheat varieties derived from the International Center for Agricultural Research in the Dry Areas
Source: Front Plant Sci. 2024 Apr 9;15:1377253. doi: 10.3389/fpls.2024.1377253 (PMC11035757; doi:10.3389/fpls.2024.1377253)
Supplement: Supplementary file 1 [file Table_2.docx]

Supp-1 Pedigree information of 159 ICARDA wheat

| Varieties | Pedigree |
| --- | --- |
| ICARDA001 | WEAVER/WL 3928//SW 89.3064/3/SOMAMA-3 |
| ICARDA002 | SERI.1B*2/3/KAUZ*2/BOW//KAUZ/4/PFAU/MILAN |
| ICARDA003 | YMI #6/GEN//TIA.1/3/VEE#5//DOVE/BUC/4/ASFOOR-4 |
| ICARDA004 | WEAVER/WL 3928//SW 89.3064/3/LAKTA-7 |
| ICARDA005 | SERI.1B*2/3/KAUZ*2/BOW//KAUZ/4/KAUZ/FLORKWA-1 |
| ICARDA006 | PBW343*2/KUKUN//22SAWSN - 97 |
| ICARDA007 | CHAMARAN/LAKTA-7 |
| ICARDA008 | ATENA-1//MILAN/DUCULA |
| ICARDA009 | SERI.1B*2/3/KAUZ*2/BOW//KAUZ/6/LFN/II58.57//PRL/3/HAHN/4/KAUZ/5/KAUZ |
| ICARDA010 | HUBARA-13/4/TRAP#1/BOW//PFAU/3/MILAN |
| ICARDA011 | PASTOR-2/3/SHUHA-7//SERI 82/SHUHA'S' |
| ICARDA012 | HUBARA-1/5/CHEN/AEGILOPS SQUARROSA (TAUS)//BCN/3/VEE#7/BOW/4/PASTOR |
| ICARDA013 | SERI.1B//KAUZ/HEVO/3/AMAD/4/SHUHA-7//SERI 82/SHUHA'S' |
| ICARDA014 | TEVEE-11/SHUHA-19/7/KEA/TAN/4/TSH/3/KAL/BB//TQFN/5/WL7168/6/SNB |
| ICARDA015 | SERI.1B//KAUZ/HEVO/3/AMAD/4/ATTILA//PSN/BOW/3/ATTILA |
| ICARDA016 | SERI.1B//KAUZ/HEVO/3/AMAD/4/MNCH/3*BCN |
| ICARDA017 | KAUZ'S'/SERI/4/SERI.1B*2/3/KAUZ*2/BOW//KAUZ |
| ICARDA018 | P1.861/RDWG//DAJAJ-10 |
| ICARDA019 | VEE7/KAUZ//PFAU/MILAN |
| ICARDA020 | SERI.1B//KAUZ/HEVO/3/AMAD/4/WEAVER/JACANA |
| ICARDA021 | SERI.1B//KAUZ/HEVO/3/AMAD/4/ESDA/SHWA//BCN |
| ICARDA022 | KAUZ'S'/SERI/3/TEVEE'S'//CROW/VEE'S' |
| ICARDA023 | KAUZ'S'/SERI/3/TEVEE'S'//CROW/VEE'S' |
| ICARDA024 | ATTILA*2/PBW65//PFAU/MILAN |
| ICARDA025 | ATTILA*2/PBW65//PFAU/MILAN |
| ICARDA026 | WHEATEAR/22SAWSN - 156 |
| ICARDA027 | YMI #6/GEN//TIA.1/3/VEE#5//DOVE/BUC/4/MILAN/PASTOR |
| ICARDA028 | TRACHA-2/SHUHA-3//KAUZ/FLORKWA-1 |
| ICARDA029 | SERI.1B*2/3/KAUZ*2/BOW//KAUZ/4/TEVEE'S'/BOBWHITE #1 |
| ICARDA030 | SERI.1B*2/3/KAUZ*2/BOW//KAUZ/4/TEVEE'S'/BOBWHITE #1 |
| ICARDA031 | DEBEIRA/4/KAUZ//ALTAR 84/AOS/3/KAUZ |
| ICARDA032 | KAUZ/SAMAR-15//RDWG/MILAN |
| ICARDA033 | CHILERO-1/4/VEE'S'/3/HORK/4MH//KAL-BB/5/CATBIRD-10 |
| ICARDA034 | HAMAM-2/FLAG-4 |
| ICARDA035 | STAR*3/LOTUS-5/4/TAM200/TUI//MILAN/KAUZ/3/CROC-1/AE.SQUARROSA (224)//OPATA |
| ICARDA036 | VEE/PJN//2*KAUZ/3/MILAN/DUCULA |
| ICARDA037 | SERI.1B*2/3/KAUZ*2/BOW//KAUZ/4/PFAU/MILAN |
| ICARDA038 | SHUHA-4//NS732/HER/3/MILAN/DUCULA |
| ICARDA039 | SHUHA-4//NS732/HER/3/MILAN/DUCULA |
| ICARDA040 | HADIAH-14/3/MUNIA/CHTO//MILAN |
| ICARDA041 | GIZA-164//TNMU/MILAN |
| ICARDA042 | GIZA-164//TNMU/MILAN |
| ICARDA043 | SERI.1B//KAUZ/HEVO/3/AMAD/4/KAUZ'S'/FLORKWA-1 |
| ICARDA044 | SERI.1B*2/3/KAUZ*2/BOW//KAUZ/4/KAUZ/FLORKWA-1 |
| ICARDA045 | SERI.1B*2/3/KAUZ*2/BOW//KAUZ/4/KAUZ/FLORKWA-1 |
| ICARDA046 | SERI.1B*2/3/KAUZ*2/BOW//KAUZ/4/KAUZ/FLORKWA-1 |
| ICARDA047 | OPATA/RAYON//KAUZ/3/PFAU/MILAN |
| ICARDA048 | SHUHA-4//NS732/HER/3/TNMU/MILAN |
| ICARDA049 | SHUHA-4//NS732/HER/3/TNMU/MILAN |
| ICARDA050 | ATTILA 50Y//ATTILA/BCN/3/PFAU/MILAN |
| ICARDA051 | JAWAHIR-1/GIRWILL-5 |
| ICARDA052 | SEKSAKA-7//SHUHA-3/PGO/SERI 82 |
| ICARDA053 | KBG-01/TOWPE |
| ICARDA054 | SIDS-1//ATTILA*2/RAYON |
| ICARDA055 | SIDS-1//ATTILA*2/RAYON |
| ICARDA056 | GIZA-168/4/ATTILA*2/3/KAUZ*2/TRAP//KAUZ |
| ICARDA057 | GIZA-168/4/ATTILA*2/3/KAUZ*2/TRAP//KAUZ |
| ICARDA058 | GIZA-168/4/ATTILA*2/3/KAUZ*2/TRAP//KAUZ |
| ICARDA059 | ATTILA*2/RAYON//CATBIRD-1 |
| ICARDA060 | ATTILA*2/RAYON//CATBIRD-1 |
| ICARDA061 | ATTILA*2/CROW/3/VEE#5/SARA//DUCULA |
| ICARDA062 | ATTILA*2/CROW/3/VEE#5/SARA//DUCULA |
| ICARDA063 | SERI.1B//KAUZ/HEVO/3/AMAD/4/HXL8246/KAUZ |
| ICARDA064 | SERI.1B//KAUZ/HEVO/3/AMAD/4/HXL8246/KAUZ |
| ICARDA065 | SERI.1B//KAUZ/HEVO/3/AMAD/4/HXL8246/KAUZ |
| ICARDA066 | SERI.1B//KAUZ/HEVO/3/AMAD/4/KAUZ/GYS//KAUZ |
| ICARDA067 | SERI.1B//KAUZ/HEVO/3/AMAD/4/KAUZ/GYS//KAUZ |
| ICARDA068 | SERI.1B//KAUZ/HEVO/3/AMAD/4/PFAU/MILAN |
| ICARDA069 | SERI.1B//KAUZ/GEN/3/AMAD/4/TEVEE'S'/SHUHA'S' |
| ICARDA070 | SERI.1B*2/3/KAUZ*2/BOW//KAUZ/4/HUBARA-13 |
| ICARDA071 | SERI.1B*2/3/KAUZ*2/BOW//KAUZ/4/HUBARA-13 |
| ICARDA072 | VEE/PJN//2*KAUZ/3/SHUHA-4/FOW-2 |
| ICARDA073 | ATTILA//VEE#5/DOBUC'S'/3/WATAN-7 |
| ICARDA074 | ATTILA//VEE#5/DOBUC'S'/3/WATAN-7 |
| ICARDA075 | ATTILA//VEE#5/DOBUC'S'/3/QADANFER-9 |
| ICARDA076 | HAR-1685 = ATILLA-7/REBWAH-12 |
| ICARDA077 | VEE/NAC//REBWAH-19 |
| ICARDA078 | VEE/NAC//REBWAH-19 |
| ICARDA079 | VEE/NAC//REBWAH-19 |
| ICARDA080 | SERI.1B*2/3/KAUZ*2/BOW//KAUZ/4/FLORKWA-2 |
| ICARDA081 | TILILA/MUBASHIIR-1 |
| ICARDA082 | TILILA/MUBASHIIR-1 |
| ICARDA083 | CHAM-4/MUBASHIIR-9 |
| ICARDA084 | CHAM-4/MUBASHIIR-9 |
| ICARDA085 | VAGA 92/EID-6 |
| ICARDA086 | HUW 234/REBWAH-19 |
| ICARDA087 | QAFZAH-7/FLAG-4 |
| ICARDA088 | QAFZAH-23/ZEMAMRA-2 |
| ICARDA089 | QAFZAH-27/SEKSAKA-6 |
| ICARDA090 | K6295-4A/FLAG-8 |
| ICARDA091 | SERI.1B*2/3/KAUZ*2/BOW//KAUZ/4/SHIHAB-7 |
| ICARDA092 | SERI.1B*2/3/KAUZ*2/BOW//KAUZ/4/SHIHAB-7 |
| ICARDA093 | WATAN-7/SEKHRAH-2 |
| ICARDA094 | WATAN-7/SEKHRAH-2 |
| ICARDA095 | PASTOR-5/SHIHAB-5 |
| ICARDA096 | MILAN/DUCULA//AL-ZEHRAA-1 |
| ICARDA097 | PVN//KAUZ/PVN/4/CROC1/AE.SQUARROSSA(205)//KAUZ/3/ATTILA |
| ICARDA098 | CROC1/AE.SQUARROSSA(205)//KAUZ/3/ATTILA/4/FLAG-1 |
| ICARDA099 | CROC-1/AE.SQUARROSA (224)//OPATA/3/FLAG-7 |
| ICARDA100 | CROC-1/AE.SQUARROSA (224)//OPATA/3/FLAG-7 |
| ICARDA101 | AMIR-2/TAJAN |
| ICARDA102 | CHIL/CHUM18//ATTILA*2/RAYON |
| ICARDA103 | KAUZ//MON/CROW'S'/3/SHUHA-4//NS732/HER/4/MILAN/PASTOR |
| ICARDA104 | SERI.1B*2/3/KAUZ*2/BOW//KAUZ/4/REBWAH-13/5/FLAG-8 |
| ICARDA105 | SERI.1B*2/3/KAUZ*2/BOW//KAUZ/4/KAUZ/GYS//KAUZ/5/MUNIA/ALTAR 84//MILAN |
| ICARDA106 | SERI.1B*2/3/KAUZ*2/BOW//KAUZ/4/KAUZ/GYS//KAUZ/5/ICARDA-SRRL-9 |
| ICARDA107 | SERI.1B*2/3/KAUZ*2/BOW//KAUZ/6/LFN/II58.57//PRL/3/HAHN/4/KAUZ/5/KAUZ/7/SITE/MO/3/VORONA/BAU//BAU |
| ICARDA108 | KAUZ/AA//KAUZ/3/SOMAMA-3/4/WATAN-10 |
| ICARDA109 | SERI.1B*2/3/KAUZ*2/BOW//KAUZ/4/ANGI-1/5/KABOWSH-1 |
| ICARDA110 | GOUBARA-1/ANGI-1//QAFZAH-21 |
| ICARDA111 | VEE7/KAUZ/3/KAUZ//MON/CROW'S'/4/QAFZAH-33 |
| ICARDA112 | SERI.1B//KAUZ/HEVO/3/AMAD*2/4/SARA 1/STAR//SW89.3064 |
| ICARDA113 | SERI.1B//KAUZ/HEVO/3/AMAD*2/4/SARA 1/STAR//SW89.3064 |
| ICARDA114 | QIMMA-12/REBWAH-13/3/NG8675/CBRD//MILAN |
| ICARDA115 | STAR*3/LOTUS-5/3/CHUM//7*BCN/4/FLAG-2 |
| ICARDA116 | STAR*3/LOTUS-5/3/CHUM//7*BCN/4/FLAG-2 |
| ICARDA117 | SERI.1B//KAUZ/HEVO/3/AMAD/4/TNMU/MILAN/5/WATAN-12 |
| ICARDA118 | HUBARA-1/5/CHEN/AEGILOPS SQUARROSA (TAUS)//BCN/3/VEE#7/BOW/4/PASTOR/6/WATAN-5 |
| ICARDA119 | HUBARA-1/5/CHEN/AEGILOPS SQUARROSA (TAUS)//BCN/3/VEE#7/BOW/4/PASTOR/6/WATAN-5 |
| ICARDA120 | HUBARA-1/5/CHEN/AEGILOPS SQUARROSA (TAUS)//BCN/3/VEE#7/BOW/4/PASTOR/6/WATAN-5 |
| ICARDA121 | HUBARA-1/5/CHEN/AEGILOPS SQUARROSA (TAUS)//BCN/3/VEE#7/BOW/4/PASTOR/6/WATAN-5 |
| ICARDA122 | P1.861/RDWG//KAPSW/SHUHA-17/3/MUBASHIIR-12 |
| ICARDA123 | KAUZ//MON/CROW'S'/3/KAUZ//KAUZ/STAR/5/SHAMIEKH-7 |
| ICARDA124 | KASYON/GENARO 81//TEVEE-1/../4/CHEN/AEGILOPS SQUARROSA (TAUS)//BCN/3/KAUZ/5/FLAG-8 |
| ICARDA125 | SERI.1B//KAUZ/HEVO/3/AMAD/4/SHUHA-7//SERI 82/SHUHA'S'/5/OPATA/RAYON//KAUZ |
| ICARDA126 | SERI.1B//KAUZ/HEVO/3/AMAD/4/PYN/BAU//MILAN/5/OPATA/RAYON//KAUZ |
| ICARDA127 | HOOSAM-8//CHAM-6/FLORKWA-2/3/ICARDA-SRRL-3 |
| ICARDA128 | HOOSAM-8//CHAM-6/FLORKWA-2/3/ICARDA-SRRL-3 |
| ICARDA129 | SERI.1B//KAUZ/HEVO/3/AMAD/4/ATTILA//PSN/BOW/3/ATTILA/5/KAUZ'S'/SHUHA-15 |
| ICARDA130 | SERI.1B//KAUZ/HEVO/3/AMAD/4/ATTILA//PSN/BOW/3/ATTILA/5/KAUZ'S'/SHUHA-15 |
| ICARDA131 | SERI.1B//KAUZ/HEVO/3/AMAD/4/ATTILA//PSN/BOW/3/ATTILA/5/KAUZ'S'/SHUHA-15 |
| ICARDA132 | SERI.1B//KAUZ/HEVO/3/AMAD/4/ATTILA//PSN/BOW/3/ATTILA/5/KAUZ'S'/SHUHA-15 |
| ICARDA133 | SERI.1B//KAUZ/HEVO/3/AMAD/4/ATTILA//PSN/BOW/3/ATTILA/5/KAUZ'S'/SHUHA-15 |
| ICARDA134 | SERI.1B//KAUZ/HEVO/3/AMAD/4/ATTILA//PSN/BOW/3/ATTILA/5/KAUZ'S'/SHUHA-15 |
| ICARDA135 | SERI.1B//KAUZ/HEVO/3/AMAD/4/ATTILA//PSN/BOW/3/ATTILA/5/KAUZ'S'/SHUHA-15 |
| ICARDA136 | SERI.1B//KAUZ/HEVO/3/AMAD/4/ATTILA//PSN/BOW/3/ATTILA/5/KAUZ'S'/SHUHA-15 |
| ICARDA137 | SERI.1B//KAUZ/HEVO/3/AMAD*2/4/ATTILA//PSN/BOW/3/ATTILA |
| ICARDA138 | SERI.1B//KAUZ/HEVO/3/AMAD*2/4/ATTILA//PSN/BOW/3/ATTILA |
| ICARDA139 | SERI.1B//KAUZ/HEVO/3/AMAD*2/4/ATTILA//PSN/BOW/3/ATTILA |
| ICARDA140 | SERI.1B//KAUZ/HEVO/3/AMAD*2/4/ATTILA//PSN/BOW/3/ATTILA |
| ICARDA141 | SERI.1B//KAUZ/HEVO/3/AMAD*2/4/ATTILA//PSN/BOW/3/ATTILA |
| ICARDA142 | SERI.1B//KAUZ/HEVO/3/AMAD*2/4/ATTILA//PSN/BOW/3/ATTILA |
| ICARDA143 | SERI.1B//KAUZ/HEVO/3/AMAD*2/4/ATTILA//PSN/BOW/3/ATTILA |
| ICARDA144 | P1.861/RDWG//DAJAJ-10/3/MILAN/PASTOR |
| ICARDA145 | P1.861/RDWG//DAJAJ-10/3/MILAN/PASTOR |
| ICARDA146 | VEE7/KAUZ/6/LFN/II58.57//PRL/3/HAHN/4/KAUZ/5/KAUZ/7/MILAN/PASTOR |
| ICARDA147 | VEE7/KAUZ/6/LFN/II58.57//PRL/3/HAHN/4/KAUZ/5/KAUZ/7/MILAN/PASTOR |
| ICARDA148 | VEE7/KAUZ//PFAU/MILAN/3/MILAN/PASTOR |
| ICARDA149 | VEE7/KAUZ//PFAU/MILAN/3/MILAN/PASTOR |
| ICARDA150 | VEE7/KAUZ//PFAU/MILAN/3/MILAN/PASTOR |
| ICARDA151 | SERI.1B//KAUZ/HEVO/3/AMAD/4/WEAVER/JACANA/5/CROC-1/AE.SQUARROSA (224)//OPATA |
| ICARDA152 | ATTILA*2/CROW//MILAN/PASTOR/3/FLAG-6 |
| ICARDA153 | SERI.1B*2/3/KAUZ*2/BOW//KAUZ/4/KAUZ/SAMAR-15/5/ICARDA-SRRL-1 |
| ICARDA154 | SERI.1B*2/3/KAUZ*2/BOW//KAUZ/4/KAUZ/SAMAR-15/5/P1.861/RDWG/3/KAUZ//MON/CROW'S' |
| ICARDA155 | SERI.1B*2/3/KAUZ*2/BOW//KAUZ/4/KAUZ/SAMAR-15/5/P1.861/RDWG/3/KAUZ//MON/CROW'S' |
| ICARDA156 | SERI.1B*2/3/KAUZ*2/BOW//KAUZ/4/KAUZ/SAMAR-15/5/P1.861/RDWG/3/KAUZ//MON/CROW'S' |
| ICARDA157 | SERI.1B*2/3/KAUZ*2/BOW//KAUZ/4/KAUZ/SAMAR-15/5/P1.861/RDWG/3/KAUZ//MON/CROW'S' |
| ICARDA158 | QIMMA-12/5/CHEN/AEGILOPS SQUARROSA (TAUS)//BCN/3/VEE#7/BOW/4/PASTOR/6/LUCO-M/BL1133//OCI/3/WEAVER |
| ICARDA159 | KATILA-7/4/CROC-1/AE.SQUARROSA (224)//OPATA/3/PASTOR/5/PASTOR//MUNIA/ALTAR 84 |

Note: “/” represents parental hybridization once, “//” represents parental hybridization twice, and the number after “*” represents how many backcrosses the material has undergone.
