# Supplementary material for: Evaluation of stripe rust resistance and genome-wide association study in wheat varieties derived from the International Center for Agricultural Research in the Dry Areas
Source: Front Plant Sci. 2024 Apr 9;15:1377253. doi: 10.3389/fpls.2024.1377253 (PMC11035757; doi:10.3389/fpls.2024.1377253)
Supplement: Supplementary file 2 [file Table_3.docx]

Supp-2 Stripe rust resistance evaluation at seedling stage

| Number | Materials | Infection Type | | |
| --- | --- | --- | --- | --- |
|  |  | CYR32 | CYR33 | CYR34 |
| 1 | ICARDA001 | 1 | 2 | 1 |
| 2 | ICARDA002 | 8 | 7 | 9 |
| 3 | ICARDA003 | 1 | 1 | 2 |
| 4 | ICARDA004 | 0 | 1 | 9 |
| 5 | ICARDA005 | 1 | 1 | 8 |
| 6 | ICARDA006 | 0 | 2 | 1 |
| 7 | ICARDA007 | 1 | 2 | 0 |
| 8 | ICARDA008 | 0 | 1 | 9 |
| 9 | ICARDA009 | 2 | 0 | 1 |
| 10 | ICARDA010 | 0 | 0 | 2 |
| 11 | ICARDA011 | 2 | 0 | 2 |
| 12 | ICARDA012 | 0 | 1 | 3 |
| 13 | ICARDA013 | 2 | 2 | 2 |
| 14 | ICARDA014 | 1 | 2 | 8 |
| 15 | ICARDA015 | 2 | 0 | 9 |
| 16 | ICARDA016 | 2 | 1 | 1 |
| 17 | ICARDA017 | 8 | 9 | 9 |
| 18 | ICARDA018 | 1 | 2 | 0 |
| 19 | ICARDA019 | 2 | 0 | 9 |
| 20 | ICARDA020 | 0 | 0 | 2 |
| 21 | ICARDA021 | 1 | 2 | 0 |
| 22 | ICARDA022 | 1 | 1 | 2 |
| 23 | ICARDA023 | 1 | 1 | 7 |
| 24 | ICARDA024 | 1 | 2 | 2 |
| 25 | ICARDA025 | 1 | 1 | 3 |
| 26 | ICARDA026 | 2 | 1 | 1 |
| 27 | ICARDA027 | 1 | 2 | 9 |
| 28 | ICARDA028 | 1 | 1 | 2 |
| 29 | ICARDA029 | 1 | 1 | 8 |
| 30 | ICARDA030 | 0 | 2 | 2 |
| 31 | ICARDA031 | 2 | 1 | 8 |
| 32 | ICARDA032 | 0 | 1 | 9 |
| 33 | ICARDA033 | 0 | 1 | 8 |
| 34 | ICARDA034 | 1 | 1 | 9 |
| 35 | ICARDA035 | 2 | 1 | 1 |
| 36 | ICARDA036 | 0 | 1 | 2 |
| 37 | ICARDA037 | 8 | 8 | 9 |
| 38 | ICARDA038 | 1 | 1 | 9 |
| 39 | ICARDA039 | 0 | 1 | 2 |
| 40 | ICARDA040 | 2 | 1 | 1 |
| 41 | ICARDA041 | 1 | 0 | 0 |
| 42 | ICARDA042 | 2 | 1 | 2 |
| 43 | ICARDA043 | 1 | 1 | 9 |
| 44 | ICARDA044 | 1 | 2 | 1 |
| 45 | ICARDA045 | 1 | 2 | 1 |
| 46 | ICARDA046 | 2 | 1 | 9 |
| 47 | ICARDA047 | 2 | 1 | 1 |
| 48 | ICARDA048 | 1 | 1 | 0 |
| 49 | ICARDA049 | 1 | 1 | 2 |
| 50 | ICARDA050 | 2 | 2 | 1 |
| 51 | ICARDA051 | 1 | 1 | 2 |
| 52 | ICARDA052 | 2 | 1 | 0 |
| 53 | ICARDA053 | 9 | 8 | 8 |
| 54 | ICARDA054 | 1 | 1 | 9 |
| 55 | ICARDA055 | 8 | 9 | 7 |
| 56 | ICARDA056 | 2 | 3 | 2 |
| 57 | ICARDA057 | 2 | 2 | 1 |
| 58 | ICARDA058 | 0 | 1 | 1 |
| 59 | ICARDA059 | 1 | 1 | 9 |
| 60 | ICARDA060 | 1 | 1 | 3 |
| 61 | ICARDA061 | 9 | 8 | 9 |
| 62 | ICARDA062 | 1 | 1 | 2 |
| 63 | ICARDA063 | 0 | 2 | 8 |
| 64 | ICARDA064 | 1 | 1 | 9 |
| 65 | ICARDA065 | 0 | 2 | 1 |
| 66 | ICARDA066 | 1 | 2 | 8 |
| 67 | ICARDA067 | 0 | 0 | 1 |
| 68 | ICARDA068 | 1 | 1 | 8 |
| 69 | ICARDA069 | 2 | 1 | 1 |
| 70 | ICARDA070 | 1 | 2 | 9 |
| 71 | ICARDA071 | 0 | 2 | 1 |
| 72 | ICARDA072 | 1 | 2 | 1 |
| 73 | ICARDA073 | 7 | 7 | 9 |
| 74 | ICARDA074 | 1 | 1 | 0 |
| 75 | ICARDA075 | 1 | 2 | 9 |
| 76 | ICARDA076 | 2 | 0 | 1 |
| 77 | ICARDA077 | 8 | 9 | 7 |
| 78 | ICARDA078 | 7 | 8 | 8 |
| 79 | ICARDA079 | 1 | 1 | 0 |
| 80 | ICARDA080 | 1 | 0 | 9 |
| 81 | ICARDA081 | 1 | 1 | 7 |
| 82 | ICARDA082 | 1 | 2 | 9 |
| 83 | ICARDA083 | 1 | 1 | 3 |
| 84 | ICARDA084 | 1 | 2 | 0 |
| 85 | ICARDA085 | 0 | 1 | 9 |
| 86 | ICARDA086 | 1 | 2 | 8 |
| 87 | ICARDA087 | 2 | 1 | 7 |
| 88 | ICARDA088 | 2 | 1 | 1 |
| 89 | ICARDA089 | 1 | 1 | 7 |
| 90 | ICARDA090 | 2 | 2 | 7 |
| 91 | ICARDA091 | 1 | 0 | 1 |
| 92 | ICARDA092 | 2 | 1 | 9 |
| 93 | ICARDA093 | 1 | 2 | 9 |
| 94 | ICARDA094 | 1 | 1 | 9 |
| 95 | ICARDA095 | 2 | 0 | 8 |
| 96 | ICARDA096 | 1 | 1 | 9 |
| 97 | ICARDA097 | 2 | 0 | 9 |
| 98 | ICARDA098 | 1 | 3 | 2 |
| 99 | ICARDA099 | 2 | 1 | 1 |
| 100 | ICARDA100 | 1 | 1 | 1 |
| 101 | ICARDA101 | 1 | 0 | 9 |
| 102 | ICARDA102 | 2 | 1 | 7 |
| 103 | ICARDA103 | 1 | 2 | 8 |
| 104 | ICARDA104 | 2 | 1 | 9 |
| 105 | ICARDA105 | 1 | 2 | 1 |
| 106 | ICARDA106 | 1 | 3 | 9 |
| 107 | ICARDA107 | 2 | 1 | 6 |
| 108 | ICARDA108 | 2 | 0 | 8 |
| 109 | ICARDA109 | 1 | 1 | 8 |
| 110 | ICARDA110 | 1 | 2 | 8 |
| 111 | ICARDA111 | 1 | 2 | 9 |
| 112 | ICARDA112 | 1 | 1 | 2 |
| 113 | ICARDA113 | 2 | 2 | 2 |
| 114 | ICARDA114 | 3 | 2 | 8 |
| 115 | ICARDA115 | 1 | 3 | 9 |
| 116 | ICARDA116 | 1 | 2 | 1 |
| 117 | ICARDA117 | 1 | 1 | 1 |
| 118 | ICARDA118 | 3 | 3 | 8 |
| 119 | ICARDA119 | 1 | 1 | 9 |
| 120 | ICARDA120 | 1 | 1 | 9 |
| 121 | ICARDA121 | 2 | 1 | 8 |
| 122 | ICARDA122 | 0 | 1 | 8 |
| 123 | ICARDA123 | 1 | 2 | 9 |
| 124 | ICARDA124 | 1 | 2 | 2 |
| 125 | ICARDA125 | 8 | 7 | 8 |
| 126 | ICARDA126 | 1 | 3 | 3 |
| 127 | ICARDA127 | 2 | 3 | 3 |
| 128 | ICARDA128 | 8 | 8 | 8 |
| 129 | ICARDA129 | 2 | 1 | 1 |
| 130 | ICARDA130 | 1 | 1 | 2 |
| 131 | ICARDA131 | 1 | 1 | 1 |
| 132 | ICARDA132 | 1 | 2 | 1 |
| 133 | ICARDA133 | 1 | 4 | 8 |
| 134 | ICARDA134 | 2 | 1 | 1 |
| 135 | ICARDA135 | 7 | 6 | 8 |
| 136 | ICARDA136 | 1 | 2 | 7 |
| 137 | ICARDA137 | 0 | 2 | 1 |
| 138 | ICARDA138 | 1 | 2 | 8 |
| 139 | ICARDA139 | 1 | 2 | 2 |
| 140 | ICARDA140 | 2 | 1 | 1 |
| 141 | ICARDA141 | 1 | 1 | 0 |
| 142 | ICARDA142 | 2 | 2 | 2 |
| 143 | ICARDA143 | 2 | 1 | 8 |
| 144 | ICARDA144 | 1 | 2 | 2 |
| 145 | ICARDA145 | 1 | 0 | 2 |
| 146 | ICARDA146 | 0 | 3 | 3 |
| 147 | ICARDA147 | 1 | 1 | 2 |
| 148 | ICARDA148 | 1 | 1 | 2 |
| 149 | ICARDA149 | 7 | 9 | 9 |
| 150 | ICARDA150 | 1 | 0 | 2 |
| 151 | ICARDA151 | 1 | 1 | 8 |
| 152 | ICARDA152 | 1 | 2 | 9 |
| 153 | ICARDA153 | 2 | 1 | 0 |
| 154 | ICARDA154 | 2 | 1 | 8 |
| 155 | ICARDA155 | 1 | 2 | 7 |
| 156 | ICARDA156 | 2 | 1 | 2 |
| 157 | ICARDA157 | 1 | 1 | 9 |
| 158 | ICARDA158 | 1 | 2 | 7 |
| 159 | ICARDA159 | 0 | 0 | 8 |

Note: “0-6” represents disease resistance, “7-9” represents susceptibility
