# Supplementary material for: Evaluation of stripe rust resistance and genome-wide association study in wheat varieties derived from the International Center for Agricultural Research in the Dry Areas
Source: Front Plant Sci. 2024 Apr 9;15:1377253. doi: 10.3389/fpls.2024.1377253 (PMC11035757; doi:10.3389/fpls.2024.1377253)
Supplement: Supplementary file 3 [file Table_4.docx]

Supp-3 Stripe rust resistance evaluation at adult-plant stage

| Number | Materials | Xining | | Guide | |
| --- | --- | --- | --- | --- | --- |
|  |  | 2022(E1) | 2023(E3) | 2022(E2) | 2023(E4) |
| 1 | ICARDA001 | R5 | R10 | R1 | R5 |
| 2 | ICARDA002 | R1 | R20 | R1 | R10 |
| 3 | ICARDA003 | R5 | R10 | R5 | R5 |
| 4 | ICARDA004 | R5 | S20 | R10 | S30 |
| 5 | ICARDA005 | MR5 | R20 | MR5 | R20 |
| 6 | ICARDA006 | R10 | R5 | R10 | R10 |
| 7 | ICARDA007 | MR5 | MR20 | MR20 | MR30 |
| 8 | ICARDA008 | R5 | S80 | R5 | S60 |
| 9 | ICARDA009 | R10 | R10 | R10 | R5 |
| 10 | ICARDA010 | R5 | R10 | R10 | R10 |
| 11 | ICARDA011 | R5 | R10 | R1 | R20 |
| 12 | ICARDA012 | R1 | R20 | R1 | R30 |
| 13 | ICARDA013 | R10 | MR30 | R10 | MR40 |
| 14 | ICARDA014 | R1 | S80 | R5 | S100 |
| 15 | ICARDA015 | R10 | S90 | R10 | S100 |
| 16 | ICARDA016 | R1 | R20 | R10 | R20 |
| 17 | ICARDA017 | S90 | S80 | S100 | S100 |
| 18 | ICARDA018 | R20 | MR10 | R10 | MR20 |
| 19 | ICARDA019 | MR30 | S100 | MR40 | S90 |
| 20 | ICARDA020 | R5 | MR30 | R1 | MR40 |
| 21 | ICARDA021 | R1 | R5 | R5 | R10 |
| 22 | ICARDA022 | R5 | R30 | R1 | R20 |
| 23 | ICARDA023 | R5 | S80 | R10 | S90 |
| 24 | ICARDA024 | R10 | MR20 | R1 | MR30 |
| 25 | ICARDA025 | R20 | R30 | R10 | R20 |
| 26 | ICARDA026 | MR20 | R5 | MR5 | R10 |
| 27 | ICARDA027 | R10 | S90 | R5 | S100 |
| 28 | ICARDA028 | R10 | R30 | R1 | R20 |
| 29 | ICARDA029 | R1 | S80 | R5 | S100 |
| 30 | ICARDA030 | R5 | R5 | R5 | R10 |
| 31 | ICARDA031 | R20 | S90 | MR40 | S90 |
| 32 | ICARDA032 | R10 | S80 | R1 | S90 |
| 33 | ICARDA033 | R10 | S80 | R10 | S90 |
| 34 | ICARDA034 | R1 | S100 | R1 | S100 |
| 35 | ICARDA035 | MR20 | MR30 | MR40 | MR10 |
| 36 | ICARDA036 | R1 | R10 | R10 | R20 |
| 37 | ICARDA037 | R10 | R20 | R10 | R10 |
| 38 | ICARDA038 | MR10 | S70 | R10 | S80 |
| 39 | ICARDA039 | R5 | R5 | R5 | R5 |
| 40 | ICARDA040 | MR5 | R5 | MR5 | R5 |
| 41 | ICARDA041 | R5 | R1 | R5 | R1 |
| 42 | ICARDA042 | MR5 | R5 | R5 | R5 |
| 43 | ICARDA043 | R20 | S60 | R20 | S60 |
| 44 | ICARDA044 | MR5 | R5 | R5 | R5 |
| 45 | ICARDA045 | R15 | R5 | R15 | R5 |
| 46 | ICARDA046 | R5 | S90 | R5 | S80 |
| 47 | ICARDA047 | R1 | R10 | R5 | R10 |
| 48 | ICARDA048 | MR10 | MR10 | MR5 | MR10 |
| 49 | ICARDA049 | R1 | R20 | R10 | MR30 |
| 50 | ICARDA050 | R5 | R10 | R1 | MR10 |
| 51 | ICARDA051 | MR10 | MR10 | MR20 | MR10 |
| 52 | ICARDA052 | R1 | R10 | R1 | R10 |
| 53 | ICARDA053 | R10 | R5 | R1 | R20 |
| 54 | ICARDA054 | R20 | S60 | R20 | S80 |
| 55 | ICARDA055 | S80 | S90 | S100 | S90 |
| 56 | ICARDA056 | R5 | MR10 | R5 | MR20 |
| 57 | ICARDA057 | R1 | R10 | R1 | R20 |
| 58 | ICARDA058 | R5 | R10 | R10 | MR10 |
| 59 | ICARDA059 | MR20 | S60 | MR30 | S60 |
| 60 | ICARDA060 | R5 | R1 | R5 | R1 |
| 61 | ICARDA061 | R20 | MR10 | R10 | R1 |
| 62 | ICARDA062 | MR20 | R5 | R1 | MR20 |
| 63 | ICARDA063 | R20 | S60 | R20 | S70 |
| 64 | ICARDA064 | R1 | S90 | MR30 | S90 |
| 65 | ICARDA065 | MR5 | R5 | R5 | R5 |
| 66 | ICARDA066 | R5 | S60 | R5 | S80 |
| 67 | ICARDA067 | MR5 | R5 | MR5 | R5 |
| 68 | ICARDA068 | R5 | S60 | R5 | S80 |
| 69 | ICARDA069 | MR20 | R5 | MR30 | R5 |
| 70 | ICARDA070 | R5 | S60 | R5 | S100 |
| 71 | ICARDA071 | MR10 | R5 | MR5 | R5 |
| 72 | ICARDA072 | R5 | R1 | R5 | R20 |
| 73 | ICARDA073 | MR5 | R20 | MR5 | R10 |
| 74 | ICARDA074 | R1 | R1 | R5 | MR20 |
| 75 | ICARDA075 | MR5 | S60 | MR5 | S60 |
| 76 | ICARDA076 | R1 | S60 | R1 | S70 |
| 77 | ICARDA077 | R5 | MR20 | R5 | R10 |
| 78 | ICARDA078 | MR20 | R10 | MR10 | R5 |
| 79 | ICARDA079 | MR10 | R5 | MR10 | R5 |
| 80 | ICARDA080 | MR5 | S20 | MR5 | S20 |
| 81 | ICARDA081 | R10 | S60 | R10 | S60 |
| 82 | ICARDA082 | MR5 | S10 | MR5 | S10 |
| 83 | ICARDA083 | R5 | R1 | MR5 | MR20 |
| 84 | ICARDA084 | MR5 | R20 | MR5 | MR10 |
| 85 | ICARDA085 | R5 | S60 | R5 | S60 |
| 86 | ICARDA086 | R5 | S60 | R5 | S60 |
| 87 | ICARDA087 | R5 | S60 | R5 | S80 |
| 88 | ICARDA088 | MR20 | R1 | MR20 | R5 |
| 89 | ICARDA089 | R10 | S60 | R10 | S80 |
| 90 | ICARDA090 | R20 | S60 | R20 | S80 |
| 91 | ICARDA091 | MR5 | R5 | R5 | R5 |
| 92 | ICARDA092 | MR30 | S60 | MR10 | S60 |
| 93 | ICARDA093 | R5 | S60 | R5 | S100 |
| 94 | ICARDA094 | R10 | S90 | R10 | S80 |
| 95 | ICARDA095 | R5 | S60 | R5 | S80 |
| 96 | ICARDA096 | R20 | S40 | R20 | S50 |
| 97 | ICARDA097 | R10 | S60 | R1 | S100 |
| 98 | ICARDA098 | R5 | S100 | R5 | S60 |
| 99 | ICARDA099 | MR20 | R1 | MR30 | R5 |
| 100 | ICARDA100 | R10 | R1 | R20 | R5 |
| 101 | ICARDA101 | MR5 | S60 | MR5 | S80 |
| 102 | ICARDA102 | MR20 | S60 | MR10 | S60 |
| 103 | ICARDA103 | MR5 | S60 | MR5 | S80 |
| 104 | ICARDA104 | R5 | S60 | R5 | S100 |
| 105 | ICARDA105 | MR10 | R5 | MR20 | R5 |
| 106 | ICARDA106 | R20 | S60 | R20 | S80 |
| 107 | ICARDA107 | R5 | S60 | R5 | S60 |
| 108 | ICARDA108 | R1 | S80 | R1 | S80 |
| 109 | ICARDA109 | MR5 | S60 | MR5 | S60 |
| 110 | ICARDA110 | R5 | S60 | R5 | S80 |
| 111 | ICARDA111 | R5 | S60 | R5 | S100 |
| 112 | ICARDA112 | MR20 | R1 | MR20 | R5 |
| 113 | ICARDA113 | R5 | R10 | R1 | R10 |
| 114 | ICARDA114 | R20 | S60 | R20 | S80 |
| 115 | ICARDA115 | R5 | S60 | R5 | S100 |
| 116 | ICARDA116 | R10 | R5 | MR20 | R5 |
| 117 | ICARDA117 | R1 | R5 | R10 | R5 |
| 118 | ICARDA118 | MR5 | S60 | MR10 | S60 |
| 119 | ICARDA119 | R20 | S80 | R20 | S100 |
| 120 | ICARDA120 | R5 | S60 | R5 | S60 |
| 121 | ICARDA121 | R5 | R1 | R5 | R1 |
| 122 | ICARDA122 | R10 | S60 | R10 | S80 |
| 123 | ICARDA123 | MR5 | S60 | MR5 | S60 |
| 124 | ICARDA124 | R50 | S60 | R50 | S60 |
| 125 | ICARDA125 | S80 | S60 | S70 | S90 |
| 126 | ICARDA126 | R5 | R10 | R5 | R10 |
| 127 | ICARDA127 | MR5 | R20 | MR5 | R20 |
| 128 | ICARDA128 | S90 | S60 | S90 | S100 |
| 129 | ICARDA129 | R10 | R10 | R10 | R20 |
| 130 | ICARDA130 | MR20 | R20 | MR20 | MR30 |
| 131 | ICARDA131 | R1 | R1 | R5 | MR20 |
| 132 | ICARDA132 | R1 | R10 | R5 | R10 |
| 133 | ICARDA133 | R1 | S60 | R1 | S60 |
| 134 | ICARDA134 | R1 | R10 | R1 | R20 |
| 135 | ICARDA135 | S100 | S90 | S80 | S60 |
| 136 | ICARDA136 | R50 | S60 | R20 | S60 |
| 137 | ICARDA137 | R10 | R1 | R20 | MR20 |
| 138 | ICARDA138 | R5 | S60 | R5 | S100 |
| 139 | ICARDA139 | R20 | R1 | R20 | R10 |
| 140 | ICARDA140 | R5 | R5 | R10 | R10 |
| 141 | ICARDA141 | MR30 | MR10 | MR20 | R5 |
| 142 | ICARDA142 | R1 | R10 | R1 | R10 |
| 143 | ICARDA143 | MR5 | S60 | MR5 | S80 |
| 144 | ICARDA144 | R5 | R5 | R5 | R10 |
| 145 | ICARDA145 | R20 | R1 | R20 | R10 |
| 146 | ICARDA146 | R5 | R5 | R10 | R10 |
| 147 | ICARDA147 | MR30 | MR10 | MR20 | R5 |
| 148 | ICARDA148 | R1 | R10 | R1 | R10 |
| 149 | ICARDA149 | S90 | S60 | S80 | S60 |
| 150 | ICARDA150 | R5 | R10 | R5 | R10 |
| 151 | ICARDA151 | R5 | S60 | R5 | S60 |
| 152 | ICARDA152 | R20 | S60 | R20 | S80 |
| 153 | ICARDA153 | R1 | R1 | R10 | R10 |
| 154 | ICARDA154 | R10 | S60 | R10 | S80 |
| 155 | ICARDA155 | R5 | S60 | R5 | S100 |
| 156 | ICARDA156 | R1 | R1 | R5 | R10 |
| 157 | ICARDA157 | R5 | S60 | R5 | S60 |
| 158 | ICARDA158 | MR30 | S80 | MR10 | S80 |
| 159 | ICARDA159 | R5 | S100 | R20 | S90 |

Note: "R" represents disease resistance, "MR" represents moderate disease resistance, "S" represents susceptibility, and the following numbers indicate degree.
