# Supplementary material for: Evaluation of stripe rust resistance and genome-wide association study in wheat varieties derived from the International Center for Agricultural Research in the Dry Areas
Source: Front Plant Sci. 2024 Apr 9;15:1377253. doi: 10.3389/fpls.2024.1377253 (PMC11035757; doi:10.3389/fpls.2024.1377253)
Supplement: Supplementary file 4 [file Table_5.docx]

Supp-4 Multi-effect sites

| SNP markers | Chromosomes | | Position | P value | |
| --- | --- | --- | --- | --- | --- |
|  |  |  |  | CYR32 | CYR33 |
| AX-111201796 | 1A | 3386274 | | 5.50E-05 | 4.70E-06 |
| AX-109057544 | 1D | 17011941 | | 6.01E-05 | 1.15E-05 |
| AX-108828080 | 1D | 17015113 | | 6.01E-05 | 1.15E-05 |
| AX-109875031 | 1D | 17047977 | | 6.01E-05 | 1.15E-05 |
| AX-109304848 | 1D | 17061640 | | 6.01E-05 | 1.15E-05 |
| AX-111504860 | 1D | 17123237 | | 6.01E-05 | 1.15E-05 |
| AX-110387208 | 1D | 17152066 | | 6.01E-05 | 1.15E-05 |
| AX-110748646 | 1D | 17175333 | | 6.01E-05 | 1.15E-05 |
| AX-110844522 | 1D | 17176342 | | 6.01E-05 | 1.15E-05 |
| AX-109278396 | 1D | 17236189 | | 6.01E-05 | 1.15E-05 |
| AX-110389791 | 1D | 17250147 | | 6.01E-05 | 1.15E-05 |
| AX-110026297 | 1D | 17251457 | | 6.01E-05 | 1.15E-05 |
| AX-111315580 | 1D | 17300365 | | 6.01E-05 | 1.15E-05 |
| AX-110269967 | 1D | 17477182 | | 6.01E-05 | 1.15E-05 |
| AX-109629993 | 1D | 17525991 | | 6.01E-05 | 1.15E-05 |
| AX-110380305 | 1D | 17618172 | | 6.01E-05 | 1.15E-05 |
| AX-109905162 | 2A | 693282543 | | 8.26E-05 | 1.26E-05 |
| AX-89410672 | 2B | 373760113 | | 3.60E-05 | 3.05E-06 |
| AX-109823691 | 2D | 64908254 | | 4.02E-06 | 6.44E-06 |
| AX-110974432 | 3A | 507311449 | | 7.87E-05 | 2.43E-05 |
| AX-110447030 | 3A | 507455106 | | 7.87E-05 | 2.43E-05 |
| AX-108736767 | 3A | 507457135 | | 7.87E-05 | 2.43E-05 |
| AX-89776892 | 3A | 507472871 | | 7.87E-05 | 2.43E-05 |
| AX-109989303 | 3B | 53391818 | | 5.32E-05 | 1.38E-05 |
| AX-108773007 | 3B | 54738088 | | 4.01E-05 | 2.61E-05 |
| AX-109589014 | 4A | 612639170 | | 5.46E-05 | 4.69E-06 |
| AX-110432011 | 4A | 613078092 | | 5.46E-05 | 4.69E-06 |
| AX-109305480 | 4A | 667064769 | | 6.19E-05 | 9.95E-06 |
| AX-110519009 | 4A | 667852552 | | 7.07E-05 | 1.10E-05 |
| AX-110577641 | 5A | 670543777 | | 5.49E-05 | 4.40E-06 |
| AX-110653920 | 5B | 503076633 | | 1.48E-05 | 1.58E-06 |
| AX-110502620 | 5B | 548240740 | | 8.57E-05 | 1.02E-05 |
| AX-109997800 | 5B | 597215371 | | 4.39E-05 | 1.48E-05 |
| AX-109408478 | 6B | 118028395 | | 2.12E-05 | 3.41E-06 |
| AX-89550449 | 6B | 118044540 | | 2.12E-05 | 3.41E-06 |
| AX-108733957 | 6B | 120671867 | | 7.55E-05 | 9.90E-06 |
| AX-111536663 | 6B | 120705920 | | 7.55E-05 | 9.90E-06 |
| AX-111700475 | 6B | 120821080 | | 7.17E-05 | 5.28E-06 |
| AX-110502033 | 6B | 120838286 | | 7.17E-05 | 5.28E-06 |
| AX-109940124 | 6B | 121265855 | | 7.55E-05 | 9.90E-06 |
| AX-111001967 | 6B | 121429721 | | 7.17E-05 | 5.28E-06 |
| AX-109314339 | 6B | 121532728 | | 7.17E-05 | 5.28E-06 |
| AX-110959650 | 6B | 121734787 | | 7.17E-05 | 5.28E-06 |
| AX-108782645 | 6B | 121796087 | | 7.55E-05 | 9.90E-06 |
| AX-108855625 | 6B | 121958790 | | 7.55E-05 | 9.90E-06 |
| AX-110577201 | 6B | 122094497 | | 7.17E-05 | 5.28E-06 |
| AX-109932370 | 6B | 122233005 | | 7.17E-05 | 5.28E-06 |
| AX-108868603 | 6B | 122242916 | | 7.55E-05 | 9.90E-06 |
| AX-110591994 | 6B | 122269171 | | 7.17E-05 | 5.28E-06 |
| AX-110624733 | 6B | 122288606 | | 7.17E-05 | 5.28E-06 |
| AX-111648853 | 6B | 122388532 | | 7.55E-05 | 9.90E-06 |
| AX-110671936 | 6B | 122675472 | | 7.55E-05 | 9.90E-06 |
| AX-109398995 | 6B | 122773344 | | 7.17E-05 | 5.28E-06 |
| AX-109815710 | 6B | 122944277 | | 7.17E-05 | 5.28E-06 |
| AX-110672473 | 6B | 123229315 | | 4.49E-05 | 7.60E-06 |
| AX-108904050 | 6B | 123300172 | | 4.49E-05 | 7.60E-06 |
| AX-109391750 | 6B | 123647957 | | 4.49E-05 | 7.60E-06 |
| AX-110079376 | 6B | 123908195 | | 4.49E-05 | 7.60E-06 |
| AX-108977840 | 6B | 136106581 | | 8.02E-05 | 7.47E-06 |
| AX-109601574 | 6B | 141174195 | | 7.62E-05 | 7.19E-06 |
| AX-109356924 | 6B | 141673340 | | 7.68E-05 | 7.37E-06 |
| AX-110987417 | 6B | 144979597 | | 7.62E-05 | 7.19E-06 |
| AX-108816205 | 6B | 150829246 | | 7.26E-05 | 9.16E-06 |
| AX-95252437 | 6D | 55925493 | | 2.74E-05 | 3.03E-06 |
| AX-86163952 | 7B | 37854718 | | 4.63E-05 | 9.70E-06 |
| AX-110962394 | 7B | 43125815 | | 7.39E-06 | 2.67E-06 |
| AX-109504466 | 7D | 38737717 | | 5.18E-05 | 4.76E-06 |
| AX-111612050 | 7D | 183355561 | | 4.08E-05 | 2.95E-06 |
