# Supplementary figures and images for: Evaluation of stripe rust resistance and genome-wide association study in wheat varieties derived from the International Center for Agricultural Research in the Dry Areas
Source: Front Plant Sci. 2024 Apr 9;15:1377253. doi: 10.3389/fpls.2024.1377253 (PMC11035757; doi:10.3389/fpls.2024.1377253)

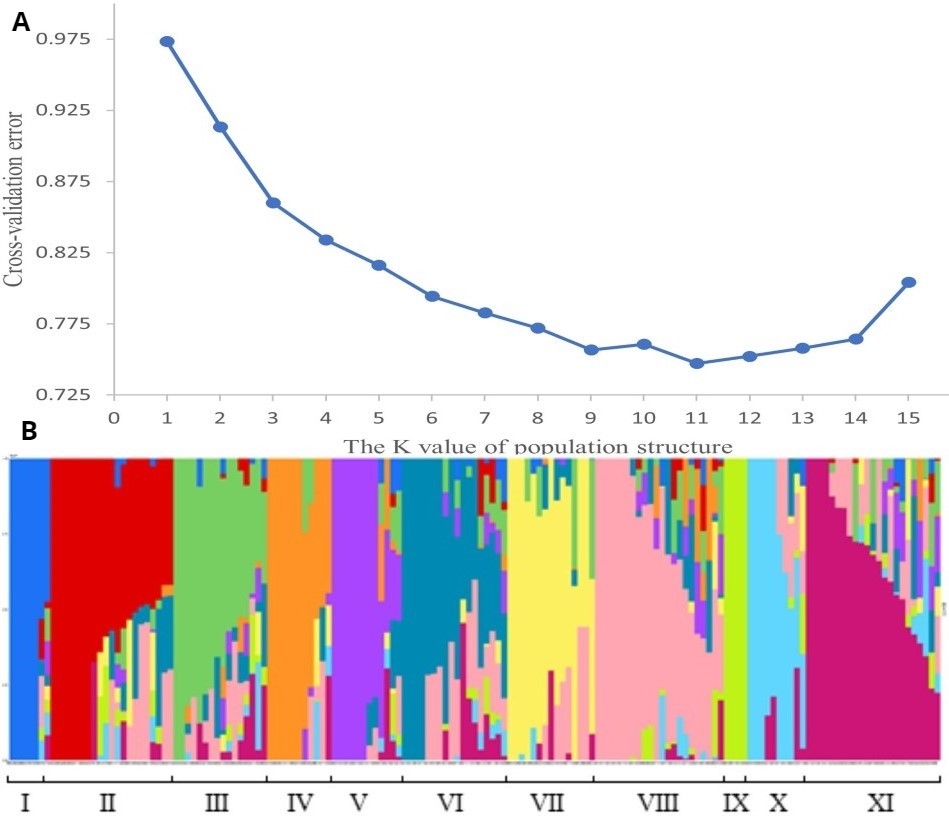

Supplement: Supplementary file 5 [file Image_3.jpeg]
